# Lessons Learned: Recommendations For Implementing a Longitudinal Study Using Wearable and Environmental Sensors in a Health Care Organization

**DOI:** 10.2196/13305

**Published:** 2019-12-10

**Authors:** Michelle L'Hommedieu, Justin L'Hommedieu, Cynthia Begay, Alison Schenone, Lida Dimitropoulou, Gayla Margolin, Tiago Falk, Emilio Ferrara, Kristina Lerman, Shrikanth Narayanan

**Affiliations:** 1 Information Sciences Institute University of Southern California Los Angeles, CA United States; 2 Department of Human Resources Keck Medicine of University of Southern California Los Angeles, CA United States; 3 Department of Psychology University of Southern California Los Angeles, CA United States; 4 Institut national de la recherche scientifique University of Québec Montreal, QC Canada

**Keywords:** research, research techniques, Ecological Momentary Assessment, wearable electronic devices

## Abstract

Although traditional methods of data collection in naturalistic settings can shed light on constructs of interest to researchers, advances in sensor-based technology allow researchers to capture continuous physiological and behavioral data to provide a more comprehensive understanding of the constructs that are examined in a dynamic health care setting. This study gives examples for implementing technology-facilitated approaches and provides the following recommendations for conducting such longitudinal, sensor-based research, with both environmental and wearable sensors in a health care setting: pilot test sensors and software early and often; build trust with key stakeholders and with potential participants who may be wary of sensor-based data collection and concerned about privacy; generate excitement for novel, new technology during recruitment; monitor incoming sensor data to troubleshoot sensor issues; and consider the logistical constraints of sensor-based research. The study describes how these recommendations were successfully implemented by providing examples from a large-scale, longitudinal, sensor-based study of hospital employees at a large hospital in California. The knowledge gained from this study may be helpful to researchers interested in obtaining dynamic, longitudinal sensor data from both wearable and environmental sensors in a health care setting (eg, a hospital) to obtain a more comprehensive understanding of constructs of interest in an ecologically valid, secure, and efficient way.

## Introduction

### Background

Facilitating a healthy and high-performing workforce has long been a topic of interest to both researchers and organizations [1] and is particularly relevant to employees working in health care settings. A significant fraction of an individual’s time is spent at work, often managing dynamic and competing cognitive, emotional, social, and physical demands. The determinants of healthy and productive work behavior and performance are, however, not well understood. Moreover, the determinants of well-being and work performance are influenced by factors outside work, and vice versa. For example, past research has found a significant relationship between employee well-being and job performance [2]. In addition, well-being is a predictor of job performance, even when controlling for job satisfaction, age, gender, and tenure [3]. Furthermore, meta analytic data show that that one’s satisfaction has a positive relationship with one’ job performance [4]. Taken together, these studies suggest that employee well-being is important to understanding job performance, and therefore more effort ought to be used to understand it.

Numerous studies examining worker wellness or job performance rely solely on self-report survey data [1,5], which are relatively easy to obtain. Such measures, however, are prone to response bias, as participants tend to underreport behaviors deemed inappropriate and overreport behaviors that are considered appropriate [6]. This bias is heightened in organizational settings, as employees often believe that their employers can see their responses or may be privy to such information in the future [6]. Consequently, obtaining accurate, dynamic data from health care employees in their workplace can pose a challenge to researchers who aim to better understand factors affecting employee wellness and workplace behaviors and outcomes.

### A Brief Overview of Sensors

Converging advances in distributed biobehavioral sensing, wireless and wearable technologies, and machine learning promise novel ways for illuminating knowledge gaps about work behavior and facilitating productive and healthy job performance in the field of health care. Wearable sensors are devices that can be worn or momentarily attached to a person’s body. They typically rely on wireless, miniature circuits embedded in patches or bandages, wristbands, rings, or shirts [7]. They can be used in tandem with hand-held devices (eg, a mobile phone) to temporarily store physiological or behavioral data and upload those data to a database server [7]. Examples of wearable sensors include smartwatches, heart rate monitors, and smart glasses [8].

Nonwearable trackers such as video cameras and gaze trackers [9] are devices that are placed in the environment and indirectly collect data from individuals. Environmental sensors are devices that are placed in the environment and collect environmental data such as temperature, humidity, and noise levels. They may pose less of a burden to participants than wearable sensors, as little to no participant interaction with nonwearable sensors is needed. However, nonwearable sensors do provide challenges for consent and privacy if data from identifiable participants are recorded without the participants’ knowledge.

### Advantages of Using Sensors in Research

Advances in sensor technology make continuous collection of behavioral and physiological data possible. Although traditional methods of data collection often yield cross-sectional data obtained at a specific point in time, both wearable and nonwearable sensors can provide a continuum of automated data [10]. The use of sensors as data collection instruments also allows researchers to overcome the limitations of traditional data collection tools, such as surveys [11], as sensor data are considered to be more objective and accurate [12,13]. Thus, sensor data can be used in tandem with self-report survey data and can help validate self-report data (eg, checking Fitbit data to identify the number of hours an individual slept the previous night and comparing this with the self-reported number of hours). Wearable sensors provide an advantage to research participants, as data can be collected unobtrusively, in real time and in natural settings. This advantage may be particularly relevant to health care employees, as they may be unable to take a break at work to complete a survey when they are busy providing critical patient care.

Sensor data can help answer diverse research questions related to working in a health care setting. Prior research has employed sensors to measure performance and personality in a postanesthesia care unit [14] and to measure physician workload to improve the safety and efficiency of emergency rooms [15]. Previous research has also used sensors to better understand employees’ mood throughout the workday [16]. Researchers in one study used the Toshiba Silmee Bar Type chest sensor to measure heart rate and pulse rate. Participants also reported their mood (ie, excited, happy, calm, tired, bored, sad, stressed, and angry) every 2 hours over 11 workdays through a mobile app called HealthyOffice. This method improved predictions of mood. Another option is the mobile sensing platform EmotionSense, developed by Jason Rentfrow and Cecilia Mascolo. EmotionSense has been used to analyze and classify participants’ voices as happy, sad, fearful, anger, and neutral [17]. Other researchers have used Moodscope, an app developed by Adrian Hosford and Caroline Ashcroft, to infer mood based on how participants used their mobile phones [18]. These researchers used text messages, emails, phone calls, app usage data, Web browsing, and location to predict mood intensity of 2 dimensions (pleasure and activeness). Sensors can also be used to examine leadership emergence and group structure [11], which may be of interest to health care leadership.

This study provides recommendations for researchers conducting sensor-based research in a health care setting. In addition, we demonstrate how we successfully implemented these recommendations and the outcomes of doing so by providing examples from a large-scale, sensor-based, longitudinal study of hospital employees. These recommendations may be useful to researchers who are interested in obtaining dynamic data in a health care setting to paint a more comprehensive picture of factors affecting job performance and worker well-being.

### Tracking Individual Performance With Sensors as a Case Study

In early 2018, we conducted the *Tracking Individual Performance with Sensors* *(TILES)* project, a large-scale study examining physiological, environmental, and behavioral variables affecting employee wellness and job performance. Over 200 volunteer hospital employees of a large hospital in Los Angeles, California, enrolled in the study; participant characteristics are described in Table 1. Participants enrolled in 1 of 3 waves of participation, each with different start and end dates. Participants in each wave were asked to wear sensors and respond to brief daily surveys for 10 weeks, starting on the first day of participation for the wave.

**Table 1:** Participant characteristics (N=212).

Demographics			Value
**Gender, n (%)**			
	Male	66 (31.1)	
	Female	146 (68.9)	
**Education, n (%)**			
	High school or some college	40 (18.9)	
	Bachelor’s degree	126 (59.4)	
	Some graduate school or graduate degree	46 (21.7)	
**Work status, n (%)**			
	Full-time	210 (99.1)	
	Part-time	2 (0.9)	
**Job title, n (%)**			
	Registered nurse	113 (54.3)	
	Certified nursing assistants	25 (12.0)	
	Monitor technicians	11 (5.3)	
	Physical therapists	6 (2.9)	
	Occupational therapists	2 (1.0)	
	Respiratory therapists	3 (1.4)	
	Other	48 (23.1)	
**Type of work, n (%)**			
	Direct patient care	155 (73.1)	
	Lab	25 (11.8)	
	Administrative	2 (0.9)	
Age (years), median (range)			36 (21-65)

**Figure 1:**
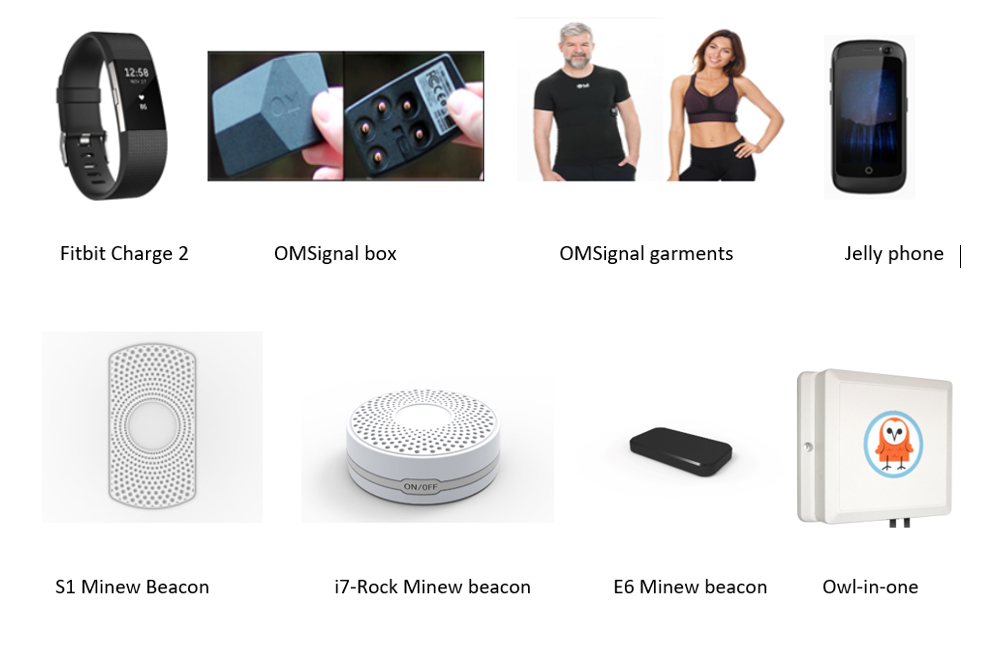
Sensors used in the TILES project. This figure includes both wearable and nonwearable sensors that we used for this study. TILES: Tracking Individual Performance with Sensors.

Participants were asked to wear multiple sensors over 10 weeks to collect physiological data including audio features, heart rate, respiratory rate, and sleep (see Table 2 for detailed descriptions; Figure 1 for images). Participants also completed survey batteries at the beginning and end of the study and daily surveys throughout the 10 weeks. Survey data were used in tandem with sensor-based data to provide a more comprehensive assessment of participant behavior.

The successful implementation of the TILES project required us to overcome a number of challenges, including identifying potential challenges with wearable sensors and making adjustments as needed, handling both the hospital’s and potential participants’ concerns regarding sensor-based data collection and privacy; garnering interest in a complex study with unfamiliar sensors; ensuring that participants were compliant with demanding, sensor-specific study procedures; and effectively implementing the study within budget and sensor manufacturer constraints. This study provides recommendations for successfully navigating such challenges based on what we learned before, during, and after study implementation.

**Table 2:** Wearable and nonwearable sensors used in the Tracking Individual Performance with Sensors (TILES) study.

Type of sensor		Description of sensor	Frequency of wear (if applicable)	Data collected from sensor
**Wearable sensors**				
	Fitbit Charge 2 (FitBit)	Wrist-worn sensor; requires companion Fitbit phone app	24 hours a day for 10 weeks	Heart rate; step count; sleep duration
	OM signal (OM signal)	Chest garment (bra for women, shirt for men) with clip-on box to collect data; requires companion OM signal app	Worn for the duration of a participant’s work shift over the course of 10 weeks	Electrocardiogram; breathing rate, depth; body motion
	Jelly phone (Unihertz)	Small Android phone that can be clipped onto participant’s clothing as a badge	Worn for the duration of a participant’s work shift over the course of 10 weeks	Audio features (eg, duration of speech and intonation)
**Nonwearable sensors**				
	S1 Minew beacon (Minew)	Environmental sensor that can be placed in different rooms; communicates with Owl-in-One sensor	No participant interaction with this sensor is required	Temperature; humidity
	i7-Rock Minew beacon (Minew)	Environmental sensor that can be attached to doors or placed in different rooms; communicates with Owl-in-One sensor	No participant interaction with this sensor is required	Motion
	E6 Minew beacon (Minew)	Environmental sensor that can be placed in different rooms; communicates with Owl-in-One sensor	No participant interaction with this sensor is required	Light level
	Owl-in-One (reelyActive)	Environmental sensor that plugs into an outlet; tracks participant proximity using Bluetooth pings from the Fitbit Charge 2	Plugged into different rooms within the hospital where participants spent most of their time; no participant interaction with these sensors was required	Participant proximity

## Methods

Efficiency, security, and ecological validity were at the forefront when planning and implementing the TILES study and are reflected in the recommendations here. Recommendations were generated in the following ways: drawing on existing knowledge of and interaction with sensors, obtaining health care leadership’s feedback on using sensors with their employees, resolving problems encountered with sensors during the study, or by post hoc reflections on what worked well and what did not. For purposes of this study, we limit our recommendations to those that relate specifically to the sensors; are believed to have the greatest impact on successful study implementation with regard to ecological validity, security, and efficiency; and can be generalized to other sensor-based research efforts.

We acknowledge that those recommendations that are grounded in the existing literature may be deemed more credible than those that are based on anecdotal challenges or post hoc reflections. Despite this, we remain confident that the strategies to overcome the challenges we encountered helped facilitate successful study implementation in a dynamic environment. The following sections outline the recommendations that were synthesized using the methods described above.

## Results

### Outcomes/Recommendations for Conducting Sensor-Based Research in a Health Care Setting

#### Recommendation 1: Pilot Test Sensors and Software Early and Often

Pilot studies are used to troubleshoot study design [19] and facilitate a smooth participant experience. In sensor-based research, conducting pilot studies can help identify whether proposed sensors are inappropriate or too complicated for participant use [19]. Sensor manufacturers may be willing to incorporate researchers’ feedback to improve upon the sensors or their companion apps. Although conducting one pilot study may be suitable for some research projects, we recommend conducting several pilot studies early on to allow ample time to test, tweak, and retest sensor procedures. This recommendation is based on the existing sensor literature [20], which highlights some of the challenges of using sensors and the benefit of testing them before deploying them in naturalistic settings.

For our study, we conducted 2 internal pilot studies in which various research team members and student volunteers wore the sensors that we planned to use in the full-scale study. We aimed to assess the comfort of the sensors, gain insight into frustration that arose from participant interaction with the sensors or software, obtain feedback about the perceived invasiveness of the sensors, test the battery life, and assess incoming data quality from the sensors. Such considerations are unique to sensor-based data collection; failing to consider these aspects of the study may result in obtaining a smaller sample size than desired, participant attrition, data sparseness, or poor data quality.

In our first pilot study, participants (n=12) wore the 3 sensors for 2 weeks. Several men found the OM signal shirt to be tight, and all the women (n=3) experienced discomfort (ie, chafing, irritation, and blisters) with the bra. We provided this feedback to the manufacturers, who provided solutions to alleviate discomfort. Pilot participants also noticed that the Jelly phones that were hung around the neck would swing. This would likely frustrate hospital employees who leaned over patients’ bedsides, so we attached a clip to the phone that could be secured onto clothing. A few participants felt uncomfortable about having their conversations potentially recorded, so we learned the importance of emphasizing that the phone was programmed to only record audio features and not content of conversations. Although this information was in our consent form, many participants expressed concerns before agreeing to participate in the pilot study and seeing the consent form. This underscores the importance of clearly articulating what data are being collected while recruiting participants, as participants will not see the consent form when they first hear about the study. The few battery issues that arose were overcome by adjusting the configuration parameters of the phone software. After making the necessary changes to sensor logistics and learning how to troubleshoot issues, we conducted another internal pilot 2 months later. Participants reported much less frustration, and the procedures were more seamless.

Before implementing the full-scale study, we conducted another pilot study within the hospital from where we would recruit study participants. A total of 6 hospital employees participated in this pilot study. During this study, we learned that participants’ familiarity with technology varied dramatically, and when difficulties with syncing sensors to their mobile phones arose, participants were, understandably, frustrated. We changed our study procedures to sync participants’ sensors to their mobile phones for them. Conducting an external pilot study allowed us to identify the challenges of conducting a sensor-based research study in a dynamic hospital environment and make the necessary adjustments to ensure a smooth participant experience.

#### Recommendation 2: Build/Maintain Trust With Those Wary of Sensors and Concerned About Privacy

Conducting a sensor-based research study in a health care setting requires researchers to effectively communicate study goals and details with both health care leadership and potential study participants. Researchers can use unique strategies tailored to each audience to build trust with those who are wary of introducing or using unfamiliar, complex sensors within the organization or concerned about the invasiveness of the sensors and how privacy may be compromised in a sensor-based study. This recommendation is grounded in the existing literature, which highlights that the acceptance of technology is an essential consideration for sensor-based data collection [21,22].

##### Build Trust Within the Organization

Building trust and developing buy-in with leadership is essential when conducting a large-scale research project in a dynamic environment [23]. The importance of doing so is heightened when employees are asked to use unfamiliar sensors that may be deemed as invasive. Developing relationships with multiple leaders and incorporating their feedback into study implementation can build trust within the health care setting and assuage leadership’s concerns about the use of sensors in an environment where privacy is of utmost importance.

Researchers are more likely to successfully conduct research in the workplace when they establish and maintain relationships with powerful organizational leaders [24]. For our study, the principal investigator leveraged an existing contact to learn about the organization’s structure and key personnel. Forming additional relationships with the organization’s leaders was crucial, as we had to ensure that the environmental sensors to be installed at the hospital would not adversely affect the hospital’s technology infrastructure such as Wi-Fi networks, power outlets, and access. We effectively leveraged our existing relationship with hospital leadership to form and maintain relationships with those who gave us feedback and permission to use sensors in the hospital.

Physiological, behavioral, and contextual data from one’s environment are sensitive in nature [25]. Leadership in a health care setting will likely have concerns about the sensors, so researchers will be tasked with assuaging such concerns [26]. Leadership will likely want to know how the research team will ensure anonymity of employee sensor data, and researchers should provide detailed information about how the sensors will be used, what data will be collected, and how those data will be used. In our study, engineers who were most knowledgeable about the sensors explained to leadership what data environmental sensors would collect (eg, temperature and humidity) and the purpose for collecting these data (eg, how does this context impact workers’ affect, behavior, and cognition?). By meeting with leaders who were knowledgeable about their field and had decision-making authority, we were able to conduct research with our sensors of choice within a sensitive environment.

##### Build Trust With Potential Participants

Obtaining high-quality data hinges on participants feeling that they can trust the researchers [26]. Wearable sensors may be perceived as more invasive than self-report surveys, thus, building trust with potential participants is important in sensor-based research. Letting health care employees know that sensor data can provide insights into the demanding nature of their jobs can be an effective way to build trust with and motivate health care employees to take on the additional tasks of learning how to use unfamiliar sensors, charge sensors, wear sensors, and submit sensor data to the research team.

For the TILES study, we attempted to build trust with potential participants by being clear about the aim of our study and emphasizing that data were collected for research purposes only. Furthermore, we were sure to note the compensation that participants would receive for participating. We met with employees and emphasized that because we know that hospital employees tend to experience high levels of stress [27] and burnout, we were studying the factors that affected their ability to thrive at work and perform well. Afterward, we introduced the novel aspect of sensor-based research and pointed out that using sensors could unobtrusively collect data to provide insights into well-being and job performance. By emphasizing that we knew the challenges of their work and highlighting the advantages of using sensors and the greater impact of our study on participants and their patients, we built trust and found common ground via a shared goal.

Informed consent is also a way to reinforce trust with participants. In our study, we crafted a consent form that thoroughly explained what was expected of participants and what data were being collected. We also clearly specified how we planned to use these data. Although this information was clearly outlined in the consent form, we learned the importance of reiterating this information to potential participants during in-person interactions with them, as some individuals may not read a lengthy, detailed consent form in its entirety.

Eliciting and responding to potential participants’ concerns regarding sensors can help build trust with potential participants, create excitement for the study, yield a larger sample size, increase compliance, and decrease attrition [28]. Researchers may ask participants to wear and interact with unfamiliar sensors that may be seen as complicated and invasive [29]. Potential participants may have questions or concerns about such sensors; therefore, researchers should be prepared to address them.

When introducing our study during our participant recruitment period, hospital employees expressed concern that the Jelly phone would record the content of conversations at work. A research team member who designed the Jelly phone software met with several employees to show them what the data from this sensor looked like and explained that the audio features in the study would be used to infer things such as stress and affect. Employees were also concerned that environmental sensor data could get back to their bosses who might ask them “What were you doing here at this time?” We clarified that location data would not be shared with hospital management and explained that environmental sensor data would allow us to estimate how much time participants spent at the bedside. We wanted to obtain an estimate of this as hospital leadership and employees said that patient care providers disliked going to medicine rooms, which decreased time spent with patients. It is important to note that this information was specified in the consent form that interested individuals gained access to after hearing about our study and expressing interest in participating. By engaging in open dialogue during the recruitment process, we appeased concerns regarding sensors, which led to participants trusting both our team and the sensors and expressing interest in participating.

#### Recommendation 3: Generate Excitement for Novel, New Technology During Recruitment

Previous attempts at longitudinal studies involving hospital employees have shown significant barriers with regard to participant recruitment and retention [30]. We were interested in learning about employee receptivity to the sensors; therefore, we took a participatory approach to recruitment. We asked directors and staff councils that comprised leaders for recommendations regarding how to initially engage and retain potential study participants, given that participants would have to learn how to use unfamiliar sensors and interact with them daily to collect sensitive data. The overarching recommendation that emerged from conversations with health care leadership was to generate excitement for the sensors used in the study, as most of this technology would be new to participants.

##### Allow Potential Participants to Interact With Sensors

We generated excitement for the sensors by having them on display at study information tables in the hospital cafeteria and by allowing passersby to interact with them and ask questions. Having team members on-site can generate initial interest in the study [31]. Employees showed interest in the OM signal sensor and Jelly phone, which were the 2 sensors that we expected them to be unfamiliar with. Upon learning about the capability of the OM signal sensor to capture breathing and respiration rate, we received positive feedback about how *cool* the sensor and accompanying app were and how it could be nice to see these data displayed on the app when exercising. Once employees had the opportunity to interact with the sensors and learn about emerging technology and its use in daily life, they seemed more excited about the prospect of joining our study to try out the sensors.

##### Personally Reach Out to Those Who May Be Wary About Sensors

Translating initial interest in the sensors into study enrollment can be challenging. Although some employees may be enticed by novel sensors and incentives for participating [28], others may become warier about participating as they learn more about the sensors and their interaction with these devices. Researchers can decrease feelings of caution by personally reaching out to these individuals.

During recruitment, we identified a gap between the number of individuals who expressed initial interest and the number of individuals who signed up for an enrollment session. We contacted these individuals and learned that many of them had questions about the sensors, such as how often they would need to wear each device and if these devices would be safe to wear inside the hospital (eg, when visiting the magnetic resonance imaging room). Although much of this information was included on our study website, we personally reached out to these individuals and enrolled many of them after answering their questions. We encourage researchers to anticipate hesitance from potential participants in sensor-based research and personally reach out to effectively translate interest into study enrollment.

##### Consider the Level of Detail Needed When Introducing Sensors

The design and content of paper recruitment materials can affect an individual’s decision to participate in a research study [32]. Researchers must balance informative yet brief content to engage the reader and must decide what sensor-related content, if any, to include. Although listing unfamiliar sensors may be meaningless or intimidating to potential participants, the enjoyment gained from learning about new products and technologies motivates people to participate in technology-related activities [33].

In our study recruitment materials, we emphasized the opportunity to be a part of a ground-breaking study that used cutting-edge technology. Paper materials contained a QR code that employees could easily scan with their mobile phone to be taken to our website to learn about the sensors. We encourage researchers to carefully consider how to best introduce the aspect of wearable sensors in their recruitment materials.

#### Recommendation 4: Monitor Incoming Sensor Data to Troubleshoot Sensor Issues

Monitoring incoming sensor data can help researchers identify technology glitches or participant issues with sensors that may otherwise go unnoticed or unreported. This recommendation is based on challenges we encountered during the study and the real-time solutions that we generated as well as post hoc reflections about what strategies worked. Not all sensors provide feedback regarding data quality and volume. In this case, researchers may have to rely on third-party quality metrics or a team member who is well versed on data analysis to determine what constitutes sensor data issues.

##### Monitoring Sensor Data to Identify Technology Glitches

Researchers should anticipate issues that may arise with wearable sensors, such as sensors breaking or companion apps crashing [34]. Participants may not be aware that a sensor or related software is malfunctioning; however, researchers may be able to identify when such an event occurs by monitoring the incoming sensor data.

The Jelly phone used in our study was initially programmed for voice activity detection to turn on the audio capture only after detecting vocal signals [35]; the battery lasted for approximately 10 to 12 hours for internal pilot testers in a university setting. While monitoring incoming Jelly data from hospital pilot participants, we noticed that we only received a couple of hours of Jelly data from a few participants. We learned that the Jelly battery died after a couple hours because of the high noise level in the hospital; thus, participants stopped wearing the Jelly. We reprogrammed the device to turn on only for a fixed duration of time (20 seconds) to save the battery. By monitoring incoming sensor data, we identified a technical glitch with our sensor and quickly developed an effective solution.

We also set up weekly calls with the manufacturers of OM signal, who provided us with usage statistics such as frequency of wear and data quality. We flagged 15.1% (32/212) of participants to check on and remeasured them to ensure appropriate sizing, checked that their companion app was collecting and sending data as expected, and reinstructed them on how to use this sensor. These issues may have otherwise gone unnoticed; therefore, we recommend that researchers monitor incoming data for both quality and volume and reach out in person to participants who fall below a predetermined threshold for both [36].

##### Monitor Sensor Data to Identify Participant Issues With Sensors

Identifying unique participant issues with sensors is important, as compliance with longitudinal research and sensors can be challenging [28,37], and researchers may ask participants to wear sensors that may be uncomfortable [38]. Monitoring incoming data allows researchers to identify sparse or low-quality sensor data, which may indicate a participant issue with the sensor, and take appropriate action [11,39].

By monitoring incoming OM signal data, we identified that several participants stopped wearing the garment, whereas several others only wore the garments for a couple of hours on each workday. We determined that 10% of participants experienced chafing, rashes, or general discomfort with the garment. For mild discomfort, we instructed participants to apply a bit of water or aloe vera to the electrodes that pressed against the skin. This solution decreased discomfort of several participants. Participants who experienced severe discomfort were instructed to refrain from wearing the garment for a few days; however, 5% continued to experience discomfort. We allowed these participants to participate without wearing the garment. We encourage researchers to anticipate that some participants may find certain wearable sensors uncomfortable and may fail to report instances of discomfort. Monitoring the incoming sensor data can help researchers identify how to accommodate participants and the unique issues they may experience with wearable sensors.

##### Monitor Sensor Data to Keep Abreast of Unresolved Issues

Depending on the sample size, number of sensors used in the study, and ease of participant sensor use, it may be challenging to identify and keep abreast of unresolved issues. Monitoring sensor data can help researchers ensure that the myriad of potential sensor issues that may arise do not go unnoticed or unresolved.

By monitoring incoming sensor data, we learned that data sparsity or low-quality data could be due to several things (eg, forgetting how to charge a sensor and not having a strong Wi-Fi connection when uploading data). We developed a shared logbook to note which participants had sparse or low-quality data so that we could follow up and assist them. We also developed a companion frequently asked questions/troubleshooting document that team members could reference to resolve sensor-related issues. Monitoring incoming data to manage and keep abreast of the different sensor issues allowed us to facilitate a smooth participant experience with the sensors.

#### Recommendation 5: Consider the Logistical Constraints of Sensor-Based Research

Researchers conducting sensor-based research will likely be constrained in their ability to purchase their most desired sensors or receive sensors in a timely manner. Although there is a seemingly endless number of constraints that may arise, budget and manufacturer constraints are 2 aspects of sensor-based research that can greatly impact the participant experience and study timeline. Thus, we urge researchers to consider the logistical constraints of sensor-based research, based on the challenges we encountered during the study, and on post hoc reflection about how we successfully navigated such challenges.

##### Consider Budget Constraints During Sensor Selection

The researcher’s budget should be carefully considered when selecting sensors to be used in data collection. Although other criteria, such as battery life and ease of participant use should also be considered during sensor selection, the suite of sensors that a researcher chooses will ultimately be constrained by their budget [40]. Having a member of the research team who is well-versed in contractual obligations or financial resources is essential.

We tested several different sensors for our study and came to our final decision based on affordability, ease of participant use, and quality of data obtained. The cost of sensors (wearable and environmental) and sensor-related products was US $233,000 (US $1142 per participant; see Table 3). Each participant received 1 Fitbit Charge 2, 1 OM signal sensor, and 1 Jelly phone; however, we purchased extra sensors to accommodate sensors malfunctioning or breaking. In addition, we purchased a surplus of OM signal garments as we did not know the accurate size breakdown of our participants until after they enrolled in the study. Additional sensor-related costs included US $5000 for charging hubs and charging cables to make charging various sensors easy for participants and US $13,000 for 64 iPod touches to accommodate Android phone users who could not access the OM signal app without an Apple device. Our project manager who was well versed in our study budget was essential to the sensor selection and purchasing process.

Researchers should also consider the cost of incentivizing participants to wear sensors. The compensation for complying with sensor-related study procedures may be influenced by several factors including how often participants are asked to wear the sensor, how comfortable the sensor is, and ease of participant interaction with the sensor [36]. For this study, structuring these incentives to be motivating enough to encourage compliance, while staying within budget, was crucial.

**Table 3:** Cost of sensors.

Sensor	Quantity, n	Unit price (US $)	Total price (US $)	Total/person (n=204) (US $)
OM signal 250 boxes and 1250 garments	250	429	107,250	525.74
Jelly phones	250	138	34,500	169.12
Owl-in-one: Bluetooth data hub and Bluetooth device proximity sensor	261	155	40,455	198.31
Minew beacons (Bluetooth environment data sensors)	139	16	2224	10.90
Fitbit Charge 2	250	124	31,000	151.96
Total	—^a^	—	215,429	1056

^a^Not applicable.

We used recommendations from Lawler [41] to guide the development of our incentive system. We made it clear what behaviors were required to obtain the reward, and we made the incentives desirable to participants [41]. For example, participants received US $75 for completing an in-person enrollment session, during which they received their sensors and instructions for use. We also paid participants between US $10 to US $25 in a prepaid Visa gift card each week based on their level of weekly compliance in wearing sensors and answering surveys. To determine compensation for each week of participation, we generated a list of actions they could do for each day and allotted a specific number of points to each action. At the end of the study, we distributed grand prizes to the top 3 participants in each wave who earned the most points throughout the 10 weeks (ie, US $250, US $200, and US $150, respectively). Such incentives highlight the magnitude of investment that may be necessary to conduct a sensor-based study, so we encourage researchers to consider the cost of incentivizing participants to interact with sensors while planning a sensor-based study.

##### Consider Sensor Manufacturer Constraints

Researchers should anticipate constraints from sensor manufacturers when planning and conducting a sensor-based study. Manufacturers may run into production delays and may be unable to deliver sensors in accordance with the expected shipment schedule [42]. These events may delay the research team’s timeline; therefore, constant communication and transparency with sensor manufacturers are crucial to the success of a sensor-based study.

Developing good rapport with sensor manufacturers helped us navigate the constraints of sensor-based research. The nature of our study required us to have all wearable sensors in hand before the study began. The OM signal garments were made to order and required 6 to 8 weeks of manufacturing and shipping time, which was challenging to navigate with a tight timeline and budget approval delays. In addition, batch ordering hundreds of sensors and receiving them on time was an obstacle, as the manufacturers were unable to process new orders during holidays. Finally, there were times when we had to exchange sensors for different sizes. When experiencing such issues, we expressed the urgency of timely delivery to the manufacturers, who accommodated us by sending sensors in partial shipments, so that we had enough sensors to cover our initial needs. We encourage researchers to delegate a few team members to establishing and maintaining relationships with sensor manufacturers, as doing so will likely help researchers navigate any manufacturer constraints that arise.

## Discussion

### Key Takeaways

Researchers will likely encounter several challenges when conducting sensor-based research. Pilot testing sensors and software early and often can help researchers iteratively improve the study procedures and user experience with sensors before the study begins. Building trust, both with key stakeholders and potential study participants who may be wary of sensor-based data collection and concerned about privacy, can aid in the recruitment of study participants and compliance with complex study procedures such as interacting with unfamiliar sensors. Researchers should aim to generate excitement for these novel sensors and technology during recruitment to spark initial interest in the study. Once the study is underway, monitoring incoming sensor data can help researchers troubleshoot issues that may have gone unnoticed. Finally, we urge researchers to consider the logistical constraints of sensor-based research. These general recommendations (summarized in Textbox 1) can be adapted to different apps of sensor technology.

**Summary of recommendations.**

1. Conduct pilot tests of sensors and software early and do so several times to anticipate what works well and what challenges may arise with the sensors to be used.
2. Build trust with health care leadership and potential participants who may be wary of sensor-based data collection and concerned about privacy.
3. Generate excitement for novel, new technology during recruitment.
4. Monitor incoming sensor data to troubleshoot sensor issues.
5. Consider the logistical constraints of sensor-based research.

### Limitations

We are confident that the recommendations put forth in this study will help facilitate a smoother investigator and participant experience with sensors. There are, however, several limitations of this study. The recommendations in this study were grounded in the existing literature, feedback from organizational leadership, challenges we encountered along the way and the solutions we generated, and post hoc reflections. This study would be strengthened with additional statistics demonstrating the effectiveness of each recommendation. These data, however, were either unavailable or challenging to quantify. For example, hospital leadership emphasized the importance of generating excitement for novel, new sensor technology rather than rattle off a list of unfamiliar sensors to participants. We recall seeing spikes in the number of participants who indicated interest on our website immediately after we spoke with them. Unfortunately, we did not keep track of the specific days and times that we spoke to participants and relayed information about “exciting” sensors; keeping such a log would have allowed us to provide descriptive statistics to speak to the effectiveness of this strategy. However, upon thoughtful reflection, feedback from participants, and seeing the number of interested individuals increase immediately after we conducted events (eg, having a study information booth in the hospital cafeteria), we are confident that this (and other strategies) was effective in having the intended impact.

We limited the recommendations in this study to those that related specifically to the sensors; were believed to have had the greatest impact with regard to ecological validity, security, and efficiency; and could be generalized to other sensor-based research efforts. Therefore, researchers should consider the recommendations we provide within the broader scope of sensor-based research and not look to this study as the sole source of recommendations for implementing such a study. For example, other sensor-specific recommendations may relate to sensor selection (eg, learning how to prioritize ease of use, battery life, data quality, and cost) or implementing strategies to encourage participant compliance with sensors.

Another limitation is that we focus on using sensors to meet a specific goal in a specific population in a specific context. Although the primary aim of this study was to provide researchers with insight into assessing overworked, shift-based employees with sensors, we acknowledge that these recommendations may be applicable and helpful to those who wish to use sensors for other purposes. To overcome this limitation, the following section discusses how these recommendations can apply to an area that is already seeing significant growth from sensor-based data collection: telemedicine/remote patient monitoring.

### Health Care Applications of Sensor Technology

Advancements in wearable sensor technology and wireless communications allow for real-time health care monitoring, which is a promising solution to treating those who live in remote areas far from medical centers or those who cannot afford inpatient care. Telemedicine, the integration of mobile communication with wearable sensors in patients, can be used to remotely monitor patients with diverse health issues including cardiac disease, diabetes, and autism spectrum disorder [43]. For example, electrodermal activity sensors, also known as skin conductance sensors, are effective for monitoring sympathetic nervous system arousal [44]. These sensors can provide those with autism spectrum disorder a means of measuring stress or anxiety when these feelings cannot be verbally or socially expressed [44]. The sensors can provide feedback to both caregivers and patients to help understand causes of stress or anxiety and help prevent negative behavior [44]. Moving toward technologies that enable continuous, outpatient monitoring is a cost-effective way to provide health care; however, health monitoring technology must be comfortable and unobtrusive, must be simple to use, and must provide privacy and security [44]. Thus, the recommendations provided in this study are applicable to the use of sensors in telemedicine.

Health care providers interested in using sensors to remotely monitor patients should pilot test sensors early and often. Pilot testing with the end user allows providers to ensure minimal discomfort to the patient and freedom of mobility, which allows the patient to carry out normal activities while under continuous monitoring. This may enhance the quality of data, as the patient’s behavior may be closer to how it is when not wearing sensors [45]. Pilot tests can also be used to test the functional aspects of sensors, such as the battery life [44,46].

Building trust with patients and generating excitement for sensors to facilitate patient health is crucial to the success of using sensors for remote health care monitoring. A patient may not believe that the sensor will give good enough signals to health care providers [45], which may result in the individual deciding not to wear the sensor as frequently as asked. Health care providers should appease patient concerns that arise with sensors, as patients may be inclined to initially reject devices [47]. Previous research identifies such concerns, including being afraid that the sensor may fall off the skin if they move too much, being afraid of wearing the sensor, being afraid that it may suddenly stop working, and worrying that it may be visible to others [45]. In addition, patients may be concerned about the security and privacy of sensor-based data [47]. In this case, health care providers could discuss a patient-centric access policy with the patient, in which different privacy levels could be assigned to the sensor data, based on sensitivity. Different access privileges could then be assigned to health care providers based on their role [47]. Informing patients about the feasibility of using sensors in their daily lives and providing instructions for use can help patients trust the health care providers and the sensors themselves. Health care providers can also highlight the advantages of continuous, sensor-based patient monitoring, such as decreased cost and less time spent going to a clinic or to the patient. Highlighting these exciting advances in technology that result in decreased patient burden can help health care providers successfully implement wearable sensors into a patient’s health care plan.

Monitoring incoming sensor data allows health care providers to help patients in real time. Sensors may have different interfaces, one for patients and one for health care providers, allowing for monitoring of incoming data. In one study, researchers employed a wearable sensor that extracted cardiac parameters such as heart rate and blood pressure to help in the early detection of diseases such as hypertension. The patient interface was the wearable sensor. This sensor extracted medical information from patients and used Bluetooth to transmit the data to an Android-based listening port, which transferred information to the Web server to show reports on the doctor interface [43]. This Web interface allowed doctors and medical centers to simultaneously view and diagnose patients’ medical status. For example, the Web interface had a module containing alarming messages (eg, signaling arrhythmia) generated by the Android-based listening port. The interface also had location records so that the doctor could track the patient’s location and send an ambulance to the patient, if needed [43]. Monitoring incoming sensor data may literally be the difference between life and death in remote patient monitoring.

Finally, we encourage health care providers to consider the logistical constraints of sensor-based patient monitoring. For example, health care providers may access sensor-based patient data for multiple patients at once [43]. In this case, the provider must consider the cost involved in obtaining sensors, teaching multiple patients how to use sensors, monitoring sensor data, and taking appropriate action (eg, calling an ambulance) during monitoring. Considering such constraints, in terms of cost and labor, will likely impact the patient experience. Sensor manufacturer constraints should also be considered when selecting sensors for remote patient monitoring. Delays in manufacturing or shipment may have a more adverse impact on the end user, as health care providers who care for critically ill patients may have less flexibility in their timeline than researchers. Being mindful of the constraints of sensor-facilitated health care can help facilitate a smoother patient monitoring experience.

### Conclusions

Advances in sensor-based technology allow for new ways of obtaining continuous physiological data from individuals in real time. Furthermore, these sensors can allow researchers to measure things that are difficult to measure with self-report and can potentially reduce the participant’s burden. Although exciting, these new methods of data collections pose new challenges to researchers and require them to adapt new skills. Nevertheless, we are confident that implementing the recommendations in this study will facilitate successful study implementation and a smooth participant experience for investigators who are interested in conducting research with sensors and will provide patient care providers with insights into how to successfully use sensors for applications such as remote patient monitoring.

## Abbreviations

IARPA: Intelligence Advanced Research Projects Activity
ODNI: Office of the Director of National Intelligence
TILES: Tracking Individual Performance with Sensors
